# Self-reported infertility and longitudinal measures of cardiovascular risk factors: The CARDIA study

**DOI:** 10.1371/journal.pone.0317867

**Published:** 2025-11-04

**Authors:** Catherine Kim, Duke Appiah, Zhe Yin, Pamela J. Schreiner, Cora E. Lewis, Megan M. McLaughlin, Adrienne N. Dula, David S. Siscovick, Heather Huddleston

**Affiliations:** 1 Departments of Medicine, Obstetrics & Gynecology, and Epidemiology, Ann Arbor, University of Michigan, Michigan, United States of America; 2 Department of Public Health, Texas Tech University Health Sciences Center, Lubbock, Texas, United States of America; 3 Department of Biostatistics, MD Anderson Cancer Center, Houston, Texas, United States of America; 4 Division of Epidemiology and Community Health, University of Minnesota, Minneapolis, Minnesota, United States of America; 5 Department of Epidemiology, University of Alabama at Birmingham, Birmingham, Alabama, United States of America; 6 Division of Cardiology, Department of Medicine, University of California, San Francisco, California, United States of America; 7 Department of Neurology, Dell Medical School at The University of Texas Austin, Austin, Texas, United States of America; 8 New York Academy of Medicine, New York City, New York, United States of America; 9 Department of Obstetrics & Gynecology, University of California, San Francisco, California, United States of America; Ankara Etlik City Hospital, TÜRKIYE

## Abstract

**Introduction:**

Previous reports have noted associations between infertility in women and increased risk of cardiovascular disease (CVD) events in later life. However, reports conflict regarding the associations between infertility and CVD risk factors. Using data from a population-based cohort of Black and White women, we examined the association between longitudinal assessments of CVD risk factors and infertility.

**Methods:**

The Coronary Artery Risk Development in Young Adults (CARDIA) study is a prospective cohort of Black and White women who have undergone repeated assessment of CVD risk factors beginning at study baseline (1985–1986). Risk factors included cigarette smoking, body mass index (BMI), blood pressure, lipid levels, glucose, and C-reactive protein. At approximately 40 years of age, an ancillary study assessed histories of infertility. We used generalized estimating equations with a logit link model to examine associations between infertility (dependent variable) and repeated CVD risk factors (independent variables), with adjustment for age, race, center, and education level in 1107 women.

**Results:**

Cigarette use and higher levels of BMI, glucose, and triglycerides and lower levels of high-density lipoprotein cholesterol (HDL) were associated with infertility after adjustment for age, race, and education. Cigarette use had the strongest associations with self-reported infertility in multivariable models (odds ratio 1.85, 95% confidence interval 1.64, 2.14).

**Conclusions:**

Women with infertility histories have adverse CVD risk factors across the reproductive lifespan, but cigarette use is the primary CVD risk factor for women’s self-reported infertility.

## Introduction

It is well-established that a history of infertility in women is associated with increased risk of cardiovascular disease (CVD) [1–4]. Possible mechanisms include adverse CVD risk factor levels among women with infertility [5]. However, the association between infertility and higher body mass index (BMI) [4–9], lipid levels [4–8], glucose [4–6,8], and inflammatory markers [10,11] is contradictory across studies. While women with higher BMI tend to have adverse CVD risk factors, risk factors are not always poor among women with infertility [4–9]. Studies may conflict due to measurement of CVD risk factors at different reproductive stages or different study inclusion criteria. Previous reports have typically measured CVD risk factors at a single point in time. However, the difference between CVD risk factor profiles among women with and without infertility may differ by age [5,12]. In addition, multiple studies of infertility examine select populations, including women seeking fertility services [13]; health professionals [1]; pregnant women [6]; and Scandinavian women [14,15]. These groups of women may have different etiologies for infertility compared to population-based studies. To our knowledge, no longitudinal reports examine the associations across the reproductive life span in a population-based study.

Our objective was to characterize the longitudinal CVD risk factor profiles of women by infertility status in the Coronary Artery Risk Development in Young Adults (CARDIA) study. CARDIA is a population-based study of CVD risk factors in Black and White women. Beginning in their early twenties, CARDIA women underwent approximately four assessments of CVD risk factors. We hypothesized that women with histories of infertility would have poorer CVD risk factor profiles, particularly cigarette use and obesity, over an extended period of time, due to the fact that infertility and CVD have these risk factors in common.

## Materials and methods

CARDIA is a multicenter longitudinal study conducted at four communities (Birmingham, Alabama; Chicago, Illinois; Minneapolis, Minnesota; and Oakland, California) to study CVD risk trends and clinical sequelae from young adulthood. Details of the study design, recruitment, methodology, and baseline characteristics are described elsewhere [16]. At baseline (Year 0 [Y0], 1985–1986), healthy adults (n = 5,115) were recruited from the general population to be balanced on sex, race (White or Black), age (18–24 or 25–30 y) and education (high school or less, or more than high school). Data collection protocols were approved by the Institutional Review Boards of each field center with all participants providing written informed consent. The ancillary CARDIA Women’s Study was conducted at exam year 16 (Y16) when women were approximately 40 years of age [17]. Women were asked, “Have you and a male partner ever had unprotected sexual intercourse for at least 12 months without becoming pregnant?” which is the phrasing used by the Centers for Disease Control and Prevention National Survey for Family Growth to account for the fact that half of pregnancies are unintended [18]. Women were also asked about reasons for infertility. Of the 1163 women who responded, we excluded 56 women due to male-factor infertility since we would not expect associations between CVD risk factors and infertility among these women. We also excluded women who reported tubal infertility, which is attributed to pelvic inflammation rather than vascular disease, for a total of 1107 participants.

CVD risk factors were assessed at baseline and approximately every 5 years [17], for approximately four assessments between about 25 years of age to 40 years of age [19]. Cigarette smoking was assessed by means of an interviewer-administered tobacco questionnaire and classified as current, former or never. BMI was calculated by dividing measured weight in kilograms by height in meters squared. Blood pressure was measured with participants seated and after 5 minutes of rest. The average of the second and third consecutive measurements was used for analysis. Glucose and lipid measurements using fasting blood samples were collected at baseline and follow-up examinations. Total cholesterol and triglyceride (TG) levels were measured enzymatically, high-density lipoprotein cholesterol (HDL-C) was determined after precipitation with dextran sulfate/magnesium chloride, and low-density lipoprotein cholesterol (LDL-C) was calculated by the Friedewald equation [20].

### Statistical analysis

Participant characteristics by infertility were defined by means (SD) or proportions as appropriate. Differences between participants in each trajectory were tested using t-tests, Wilcoxon tests, and χ2 analyses for continuous and categorical characteristics, respectively. To account for repeated measures of CVD risk factors between Y0 and Y16, we used generalized estimating equations with a logit link model to examine associations between infertility (dependent variable) and CVD risk factors (independent variables). Thus, infertility may have been present at any time under observation. Each CVD risk factor was initially evaluated in a separate model. All models adjusted for age, race, center, and education. We also evaluated a model with all of the risk factors combined. In a sensitivity analysis, we also constructed models that adjusted for household income, physical activity, and alcohol use, but the pattern of results did not change (results not shown). In another sensitivity analysis, we constructed models that adjusted for pregnancy prior to the baseline exam. Finally, we examined each race separately and saw a similar pattern to the overall results. Analyses were performed using R version 4.4.1.

## Results

Table 1 shows the characteristics of women at baseline, when they were aged approximately 25 years of age. The majority of women had been pregnant before baseline. At Y16, approximately one-third of women (n = 375) reported ever experiencing infertility. Of the 141 women who sought medical care for infertility, the most common etiology was irregular menstrual cycles (n = 35), followed by uterine abnormalities (n = 21), and endometriosis (n = 18); women could report more than one etiology. At Y0, infertile women were more likely to be black, to have less than a high school education, had a pregnancy or live birth, to smoke, to have slightly higher levels of physical activity, and to have higher BMI and lower HDL.

**Table 1:** Characteristics of women at the baseline exam (CARDIA Exam Year 0) by any history of infertility reported at Exam Year 16. Continuous variables compared using t-tests and categorical variables compared using χ^2^ tests.

	No history of infertility	History of infertility	p-value
	n = 732	n = 375	
Age (years)	25.3 (3.6)	25.1 (3.7)	0.29
Race (n, %)			<0.0001
Black (n, %)	351 (48%)	238 (64%)	
White	381 (52%)	137 (36%)	
Less than high school education (n, %)	241 (33%)	160 (43%)	0.001
Family income < $50,000/year (n, %)	526 (72%)	281 (75%)	0.28
Physical activity score (exercise units)	352.5 (258.1)	323.3 (262.7)	0.026
Alcohol use (mean ml per day)	6.3 (11.0)	9.0 (18.9)	0.073
Current cigarette use (n, %)	164 (22.4%)	133 (35.5%)	<0.0001
Any prior pregnancy (n, %)	372 (50.8%)	224 (59.7%)	0.0059
Body mass index (kg/m^2^)	24.6 (5.7)	25.6 (6.1)	0.0089
Systolic blood pressure (mmHg)	106.8 (10)	107.3 (10.3)	0.41
Diastolic blood pressure (mmHg)	67.3 (8.8)	67.3 (8.9)	0.98
Fasting glucose (mg/dL)	80.3 (12)	81.7 (19.6)	0.19
Triglycerides (mg/dL)	66.7 (35.8)	68.3 (36.3)	0.50
High-density lipoprotein cholesterol (mg/dL)	56.3 (13.1)	53.9 (11.7)	0.0019
Low-density lipoprotein cholesterol (mg/dL)	108.7 (29.2)	109.4 (30.6)	0.71

**Fig 1:**
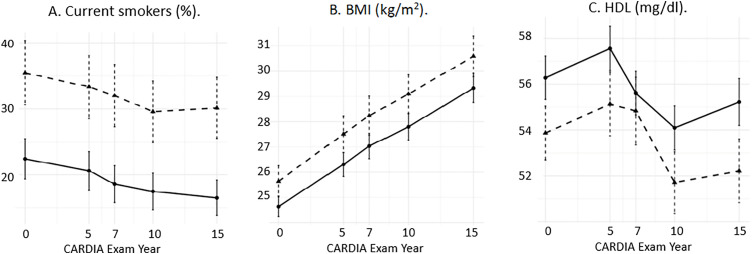
Unadjusted risk factor levels at each CARDIA Exam Year by infertility status. Women with histories of infertility are shown in the dashed lines, women without histories of infertility are shown in the solid lines. Panel A indicates proportion of current cigarette smokers, Panel B indicates mean BMI (kg/m^2^), Panel C indicates mean high-density lipoprotein (mg/dl). Point estimate and 95% confidence intervals shown.

These risk factor patterns continued into later years. At each CARDIA exam, women with infertility were more likely to smoke cigarettes, have higher mean BMI and tended to have lower HDL (Fig 1). In models adjusted for age, race, study center, and education level, several but not all CVD risk factors were associated with infertility. Cigarette use was the strongest risk factor. Associations with higher BMI and glucose levels and lower HDL were also observed (Table 2).

**Table 2:** Association between any history of infertility by Exam Year 16 (dependent variable) and cardiovascular disease risk factors (independent variables) between Exam Year 0 and Exam Year 16. Each risk factor evaluated in a separate model, and all models adjust for age, race, center, and education at baseline. Odds ratios (95% confidence intervals) shown per unit of covariate.

	Odds ratio (95% CI)	p-value
Current cigarette use	1.85 (1.64, 2.14)	<0.0001
Body mass index (kg/m^2^)	1.01 (1.0004, 1.02)	0.04
Systolic blood pressure (mmHg)	1.00 (0.997, 1.01)	0.43
Diastolic blood pressure (mmHg)	1.01 (0.999, 1.01)	0.13
Fasting glucose (mg/dL)	1.00 (1.001, 1.01)	0.01
Triglycerides (mg/dL)	1.001 (1.0001, 1.003)	0.04
High-density lipoprotein cholesterol (mg/dL)	0.99 (0.986, 0.995)	<0.0001
Low-density lipoprotein cholesterol (mg/dL)	1.001 (0.999, 1.003)	0.59
C-reactive protein (mg/L)	1.01 (0.99, 1.02)	0.43

**Fig 2:**
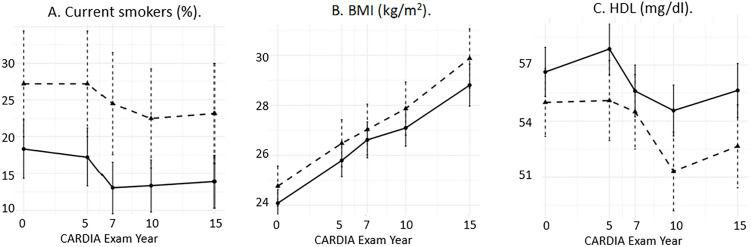
Unadjusted risk factor levels at each CARDIA Exam Year by infertility status among women who never conceived. Women with histories of infertility are shown in the dashed lines, women without histories of infertility are shown in the solid lines. Panel A indicates proportion of current cigarette smokers, Panel B indicates mean BMI (kg/m^2^), Panel C indicates mean high-density lipoprotein (mg/dl). Point estimates and 95% confidence intervals shown.

In a model with all risk factors included, smoking remained significantly associated with infertility, although the association between current smoking (yes/no) and infertility was significantly reduced (OR 1.04, 95% CI 1.02, 1.06, p-value 0.0002). BMI was no longer significant, and HDL had a significant but weak association with infertility possibly due to collinearity. These patterns did not change with additional adjustment for any pregnancy or when restricted to women who had not conceived at baseline or when restricted to women who never conceived, although the differences between groups became smaller (Fig 2).

## Discussion

In this study, cigarette smoking was the strongest risk CVD risk factor for infertility. BMI was also associated with infertility, but this association was no longer significant after adjusting for cigarette use. This suggests that abnormal CVD risk factor profiles during the reproductive age, particularly cigarette use, account for some of the associations previously reported between infertility and CVD risk [1,14,21,22]. However, aside from smoking, CVD risk factors are likely not the reason that infertility and CVD risk factors are associated. HDL was the only other risk factor associated with infertility in multivariable adjustment. This report adds to the literature by examining multiple risk factors across several decades in a population-based cohort of Black and White women. Of note, our analysis was able to account for the fact that continuous risk factor levels were typically within normal range. We also were able to examine risk factors over an extended period of time.

Although previous studies have noted that CVD risk factors and infertility are linked, studies differed as to which risk factors were most important, particularly when considered together in multivariable models. Previous studies differed in populations sampled and at what ages. Our results are consistent with prior studies that have reported abnormalities between infertility and CVD risk factor profiles performed at a single point in time, in selected populations. However, these studies were not consistent in which factors were associated with infertility. In one 2020 meta-analysis, women with histories of infertility had higher BMI, LDL, and triglycerides compared to fertile women [4]. Glucose levels were similar in women with and without infertility histories [4]. In contrast, in Project Viva, a cohort of pregnant women, midlife participants (~50 years of age) with histories of infertility (n = 160) had higher glucose levels than women without histories of infertility (n = 308) [6]. Other risk factors were statistically similar, and differences were not significant postmenopause. Mid-life women in the Framingham Heart Study Third Generation/Omni Cohort Exam who had histories of infertility (n = 282) had higher odds of obesity than women without histories of infertility (n = 1686) [8]. Otherwise, risk factors were similar by infertility [8]. Several analyses of women participants in the National Health and Nutrition Survey (NHANES), a population-based cross-sectional study, noted that BMI, glucose, and lipids were poorer among women with histories of infertility compared to women without infertility [5,7,23].

In the present report, cigarette use was the risk factor most strongly associated with infertility. The significance of other CVD risk factors was markedly reduced in multivariable models including cigarette use. In one report using NHANES data, current smoking increased the odds of infertility by almost fifty-percent, even after adjustment for other sociodemographic factors [24]. Other analyses of the same participants were notable for the lack of a graded association between tobacco use and infertility [5], suggesting that even minimal exposures to cigarettes over time increased infertility risk. Exposure to cigarettes decreases ovarian reserve [25] through toxic effects upon ovarian granulosa cells [26]. Although the adverse effects of smoking for pregnancy health and CVD are established, older studies suggest that associations with infertility are not as well-recognized [27]. Dissemination of these risks may provide an additional deterrent to cigarette use in reproductive-age women.

We also found that lower levels of HDL cholesterol were associated with infertility, even after consideration of BMI and smoking. Previous reports in NHANES have noted that higher levels of HDL are associated with lower odds of fertility [23]. Although mechanisms are speculative, HDL may serve as a substrate for key oocyte metabolites and has been linked with preimplantation embryo quality; mice with disrupted HDL metabolism have reduced fertility, which may be reversed upon restoration of cholesterol profiles [28].

Strengths of this report include the repeated measures of CVD risk factors and the population-based cohort including large numbers of Black and White women. However, this report has several limitations. Infertility was based upon self-report, and diagnostic tests addressing the etiology of fertility were not available. The definition of infertility varies; infertility has also been defined as delayed time to conception, specifically longer than six cycles [29] or alternatively as lack of response to assisted reproductive technology [29]. In this population-based study, women did not undergo diagnostic testing to characterize fertility. Thus, the findings cannot be applied to women seeking care for infertility, and the importance of CVD risk factors for women seeking care for infertility may differ by population. Of note, previous studies examining CVD risk factors in these populations have also reported the adverse impact of cigarette smoking [30] and obesity [31]. Subanalysis by etiology was not possible and examination of CVD events was not possible due to sample size.

### Limitations

Women’s recall of infertility was not ascertained at each exam. Infertility is defined as a condition rather than a state; estimates of association may reflect risk factors which changed after infertility rather than prior to infertility. In other words, the analysis does not prove that smoking leads to infertility and it is possible that smoking and other CVD risk factors followed, rather than preceded, the year of unprotected intercourse that did not result in pregnancy. Postpartum lifestyle changes and assisted reproductive technologies may have adversely influenced CVD risk factor levels. Findings may not apply to women of other races and ethnicities. The cohort was incepted approximately forty years ago, and it is possible that the CVD risk factor profiles of infertile women differ in the present day. The impact of such changes would be minimized due to repeated measures of CVD risk factors in each woman. Finally, infertility is a heterogeneous condition. These findings may not apply to populations with male or tubal factor infertility. Strength of associations may vary by cause of infertility, particularly when risk factors are collinear.

We conclude that longitudinal CVD risk factor profiles, particularly cigarette smoking and lipid levels, are associated with infertility, and may be a pathway through which infertility and CVD are linked. However, CVD risk factors are likely not the primary mechanism linking infertility and CVD. Women should be provided with information regarding optimization of risk factors, particularly smoking, for fertility as well as future CVD risk. Whether cigarette use has a greater impact upon infertility among women with no tubal factor infertility or among other subgroups such as women with fibroids or women with advanced maternal age could guide studies of interventions in these subgroups. Targeted interventions for smoking cessation medications, including nicotine replacement as well as other pharmacologic agents, could potentially be explored among pregnant women and in animal models. Adequately powered studies examining the relationship between infertility and CVD events after the reproductive stage has ended are needed.
